# NKX2-1-mediated p53 expression modulates lung adenocarcinoma progression via modulating IKKβ/NF-κB activation

**DOI:** 10.18632/oncotarget.3695

**Published:** 2015-03-30

**Authors:** Po-Ming Chen, Tzu-Chin Wu, Ya-Wen Cheng, Chih-Yi Chen, Huei Lee

**Affiliations:** ^1^ Graduate Institute of Cancer Biology and Drug Discovery, Taipei Medical University, Taipei City, Taiwan; ^2^ Division of Chest Medicine, Department of Internal Medicine, Chung Shan Medical University Hospital, Taichung, Taiwan; ^3^ Department of Surgery, China Medical University Hospital, Taichung, Taiwan

**Keywords:** NKX2-1, p53, IKKβ, lung adenocarcinoma

## Abstract

NKX2-1 plays a dual role in lung adenocarcinoma progression, but the underling mechanism is not fully understood. In the present study, we provide evidence that NKX2-1 directly regulates p53 transcription, and in turn, NKX2-1 elevates the mutant p53/NF-Y complex to up-regulate IKKβ transcription in p53-mutant cells, but NKX2-1-mediated wild-type p53 down-regulates IKKβ transcription via decreased Sp1 binding to IKKβ promoter in p53-WT cells. The IKKβ-mediated p65 nuclear localization and epithelial-to-mesenchymal transition (EMT) modulated by the NKX2-1/p53 axis is responsible for soft-agar growth, invasion, and xenograft tumour formation. Among patients, high-IKKβ mRNA tumours had higher prevalence in p53-mutant or nuclear p65 tumours than their counterparts, but not related with NKX2-1 mRNA expression. However, when tumours were divided into p53-WT and p53-mutant subgroups, NKX2-1 mRNA expression was negatively correlated with IKKβ mRNA in p53-WT subgroup, but positively related with IKKβ mRNA expression in p53-mutant subgroup. Kaplan-Meier and Cox regression analysis indicated that high NKX2-1 mRNA tumours exhibited poorer overall survival and relapse free survival than low NKX2-1 mRNA tumours in p53-WT subgroup, but the opposite was observed in p53-mutant subgroup. Therefore, we suggest that NKX2-1 as a tumour suppressor or a tumour promoter in lung adenocarcinoma progression is dependent on p53 status.

## INTRODUCTION

NKX2-1, also called thyroid transcription factor (TTF-1), is a homeodomain nuclear protein that belongs to the NKX2 family of transcription factors [1]. A large-scale project to characterise copy-number alterations in 371 primary lung adenocarcinomas indicated that amplification of chromosome 14q13.3 is the most common event and NKX2-1 was identified to lie in the minimal 14q13.3 as a novel candidate proto-oncogene [2]. Further, NKX2-1 as a lineage-survival oncogene was reported in T cell acute lymphoblastic leukemia [3] and oncogenic ROR1 and LMO3 directly targeted by NKX2-1 promote lung adenocarcinoma progression [4, 5]. Conversely, some reports showed that NKX2-1 may suppress lung adenocarcinoma progression via impeding TGF-β-induced epithelial-to mesenchymal transition (EMT) due to increased E-cadherin expression [6] and occuludin targeted by NKX2-1 impeded migration and induced aknoikis in lung cancer cells [7]. Moreover, an animal model with Kras activation combined with p53 deletion mice initiated lung adenocarcinoma development that demonstrated that NKX2-1 was only lost in malignant metastatic tumours, not in non-metastatic tumours [8]. However, haploinsufficiency of NKX2-1 enhanced KrasG12D-mediated tumour progression, but reduced EGFRL858R-mediated progression in transgenic mice model [9]. This result suggests that the dual function of NKX2-1 in lung adenocarcinoma progression might be dependent on different driver gene mutations.

The NF-κB pathway is required for the development of lung adenocarcinoma [10]. P65 nuclear translocation, resulting in IkBα phosphorylation by IKKβ, plays a crucial role in activating NF-κB signalling pathway [11-13]. Loss of p53 and expression of oncogenic Kras (G12D) have been shown to result in NF-κB activation in primary mouse embryonic fibroblasts [10]. Conversely, p53 restoration has led to NF-κB inhibition in lung tumour cell lines that expressed Kras mutations and lacked p53 [10]. In an animal model, the inhibition of the NF-κB signalling pathway in lung tumours *in vivo* resulted in significantly reduced tumour formation [14]. However, the underlying mechanism of how p53 regulates the NF-κB signalling pathway is not well understood.

P53 function is predominately regulated in post-translational levels, such as phosphorylation, acetylation, and methylation for its protein stability, but there is little information in the transcription level for regulating p53 function [15]. Surprisingly, we observed that p53 protein and mRNA expression were positively correlated with NKX2-1 expression in lung cancer cells. In the present study, we provided the evidence to demonstrate that NKX2-1-mediated p53 expression controls tumour progression in lung adenocarcinoma via modulating IKKβ/NF-κB activation.

## RESULTS

### NKX2-1 is positively correlated with expression of p53 and p21 in p53-wild-type cells, but negatively related with p21 expression in p53-mutant cells

**Figure 1:**
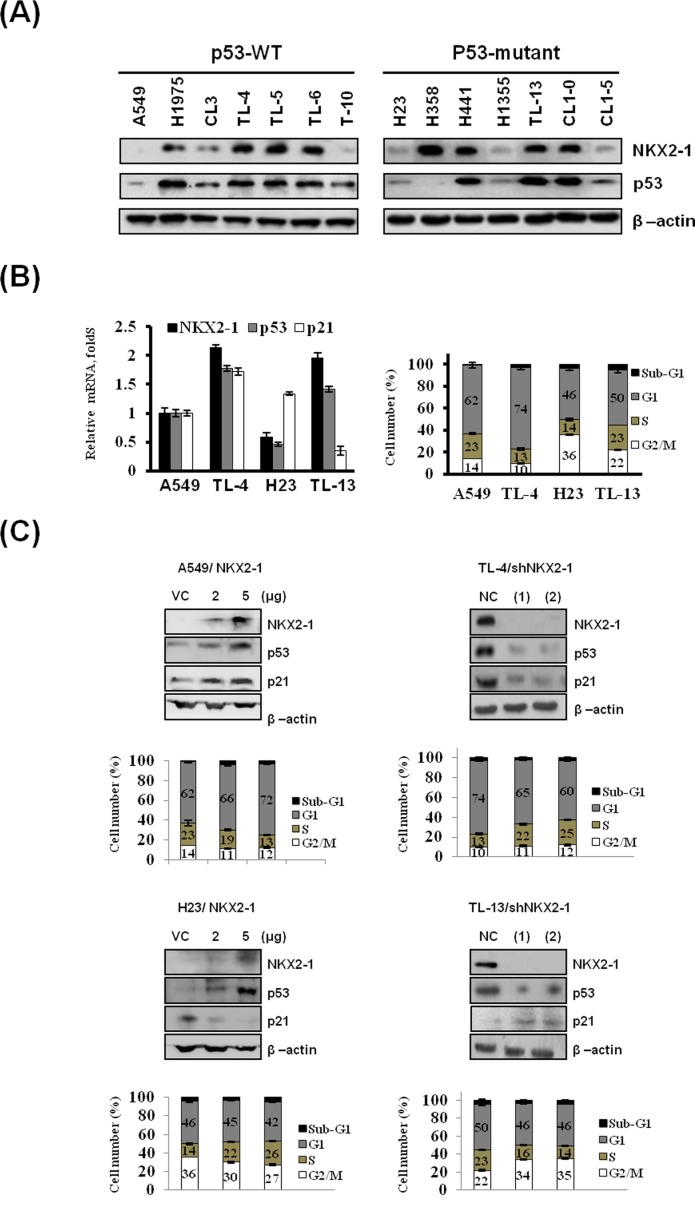
Correlation of NKX2-1 expression with p53 and p21 expression to modulate the distribution of cell cycle phase in p53-WT and -mutant lung adenocarcinoma cells (**A**) NKX2-1 and p53 protein expressions were evaluated by western blotting in a panel of lung cancer cell lines with or without p53 mutation. (**B**) The expression levels of NKX2-1, p53, and p21 mRNA in A549, TL4, H23, and TL13 cells were determined by real-time RT-PCR (left panel). The distribution of cell cycle phases of these four cells were analysed by a flow cytometry analysis and the cell number of the cell cycle phase of each cells was indicated as a percentage (right panel). (**C**) Two shRNAs were transfected into TL4 and TL13 cells to silence NKX2-1 expression and two doses of NKX2-1 expression vector were transfected into A549 and H23 cells for 48 hr and western blotting was performed to determine the expression of NKX2-1, p53, and p21 in these both cells after transfected with shRNAs or expression vector of NKX2-1 (upper panel). The distribution of cell cycle phases were determined by a flow cytometry and the cell number of the cell cycle phases changed by NKX2-1-knockdown or –overexpression were indicated as the percentage of the NC cells.

A panel of p53-wild-type (WT) and p53-mutant lung adenocarcinoma cell lines were enrolled to test whether NKX2-1 expression could be associated with p53 expression. Western blotting indicated that NKX2-1 expression was generally positively correlated with p53 expression in p53-WT and p53-mutant cells, but this association was not observed in TL-10 and H358 cells (Figure 1A). Four out of 14 cell lines were collected to determine the mRNA levels of NKX2-1, p53, and p21 using real-time RT-PCR analysis to verify whether NKX2-1 could regulate p53 transcription and consequently to modulate p53 downstream gene p21 expression. As shown in Figure 1B (left panel), p53 mRNA expression levels was positively correlated with NKX2-1 mRNA expression in p53-WT A549 and TL-4 and p53-mutant H23 and TL-13 cells. p21 mRNA expression levels were positively correlated with NKX2-1 expression in p53-WT cells, but the opposite was observed in p53-mutant cells. The distribution of G1 and S phase cells evaluated by a flow cytometry analysis can be supported the change of p53 and p21 expression by NKX2-1 in these four cells (Figure 1B right panel). In addition, two small hairpin (sh)RNAs were used to silence NKX2-1 expression in TL-4 and TL-13 cells. Western blotting indicated that the expression of NKX2-1, p53 and p21 were markedly decreased by NKX2-1 silencing using two shNKX2-1 in TL-4 and TL-13 cells (Figure 1C right upper panel). The distribution of cell cycle phase was consistent with the decrease in the expression of p53 and p21 by NKX2-1 silencing in TL-4 and TL-13 cells (Figure 1C right lower panel). The opposite in the expression of p53 and p21 and cell cycle phase were observed in NKX2-1-overexpressing A549 and H23 cells (Figure 1C left panel). These results suggest that NKX2-1 might regulate p53 transcription and then to modulate p21 expression in p53-WT and p53-mutant cells.

### NKX2-1 directly regulates p53 transcription, regardless of p53 mutational status

**Figure 2:**
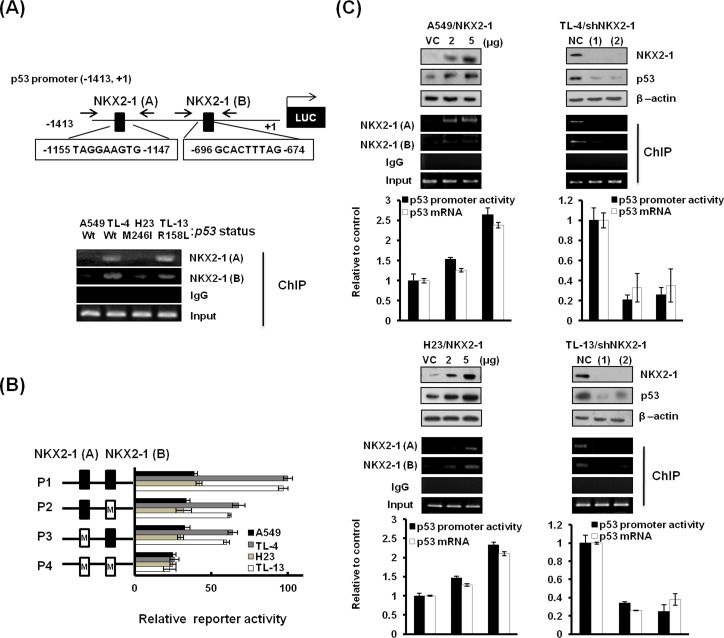
NKX2-1 directly regulates p53 transcription, regardless of p53 status (**A**) Schematic diagram of p53 promoter-driven luciferase reporters: p53 (−1413/+1)-Luc. NKX2-1(A) and NKX2-1(B) possess the two putative NKX2-1 binding sites. The binding activity of NKX2-1 to the p53 promoter was evaluated by ChIP in A549, TL-4, H23, and TL-13 cells. (**B**) The two NKX2-1 putative binding sites on the p53 (−1413/+1)-Luc were mutated by site-directed mutagenesis, and the mutated promoters were transfected into A549, TL-4, H23, and TL-13 cells to evaluate the reporter activity by luciferase reporter activity assay. (**C**) The binding activity of NKX2-1 to the p53 promoter was evaluated by ChIP and luciferase reporter activity assay and the p53 mRNA expression was evaluated by real-time RT-PCR in NKX2-1-knockdown (two shRNAs) TL-4 and TL-13, and NKX2-1-overexpressing A549 and H23 for 48 hr. NKX2-1, p53 in whole lysates of these cells were evaluated by western blotting. β-actin was used as a loading control for whole cell lysates.

Two NKX2-1 putative binding sites (−1155/−1147 and −696/−674) on the p53 promoter region (−1413/+1) were predicted by a software analysis (http://www.cbrc.jp/research/db/TFSEARCH; Figure 2A upper panel). To verify whether NKX2-1 could directly regulate p53 transcription, the p53 promoter (−1413/+1) was constructed for ChIP and luciferase reporter activity assays. ChIP analysis indicated that a higher DNA binding activity of NKX2-1 on the p53 promoter was seen in high-NKX2-1 expressing TL-4 and TL-13 cells than in low-NKX2-1 expressing A549 and H23 cells (Figure 2A lower panel). The binding activity of NKX2-1 A (−1155/−1147) on p53 promoter was greater than NKX2-1 B (−696/−674). To further investigate whether NKX2-1 could be responsible for p53 transcription, two NKX2-1 putative binding sites on the p53 promoter (−1413/+1) were mutated by site-directed mutagenesis, and four p53 promoters (P1, P2, P3, and P4) with different mutation statuses of NKX2-1 putative binding sites were constructed and then transfected into these four cells for luciferase reporter activity assay (Figure 2B). As expected, the reporter activity of these four promoters in high-NKX2-1 expressing TL-4 and TL-13 cells were markedly decreased by the mutations of NKX2-1 binding sites, but changes in the reporter activity were not observed in low-NKX2-1 expressing A549 and H23 cells (Figure 2C). A decrease of p53 protein expression in NKX2-1-knockdown TL-4 and TL-13 cells and an increase of p53 protein expression in NKX2-1-overexpressing A549 and H23 cells were consistent with their its mRNA and reporter activity activities (Figure 2C). ChIP analysis confirmed the increase or decrease of NKX2-1 binding activity on p53 promoter to be modulated by NKX2-1-knockdown or -overexpression (Figure 2C). These results clearly indicate that NKX2-1 directly regulates p53 transcription in lung adenocarcinoma cells, regardless of p53 status.

### NKX2-1-mediated p53 expression modulates soft-agar growth, invasiveness, and xenograft tumour formation

**Figure 3:**
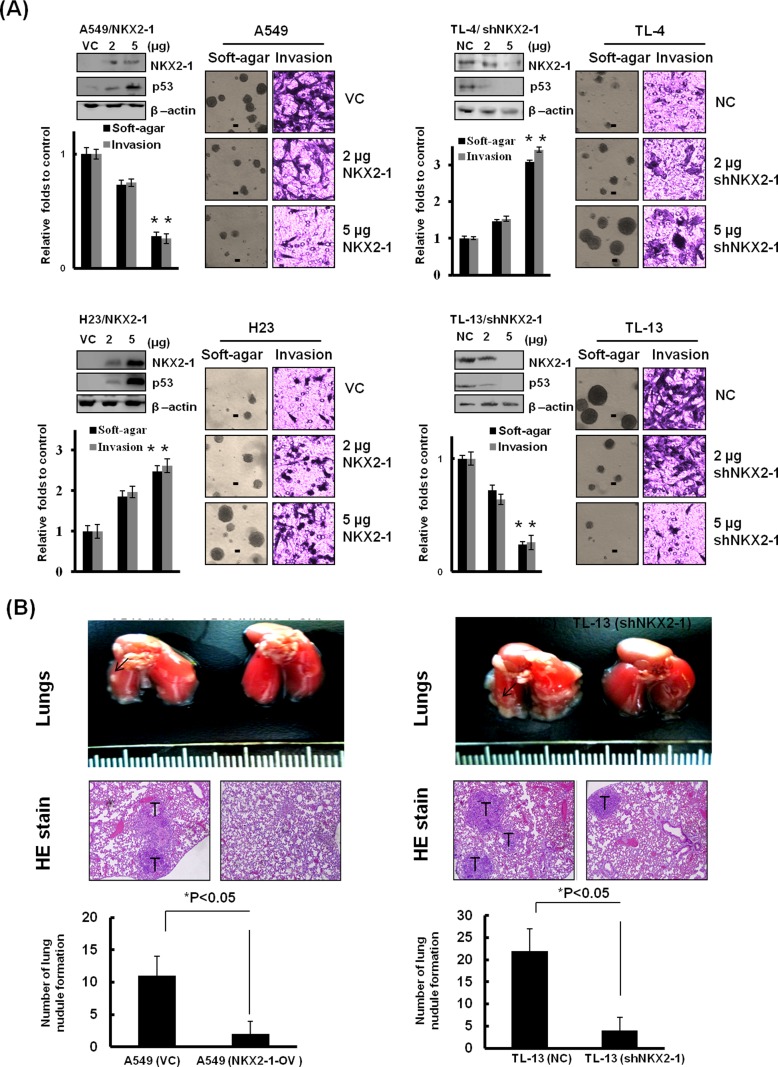
NKX2-1-mediated p53 attenuates soft-agar growth, invasiveness, and xenograft tumor formation (**A**) After a 48-hr transfection of wild-type p53 A549 and TL-4 cells and of mutated p53 TL-13 and H23 cells with indicated dosages of NKX2-1 cDNA plasmid and shNKX2-1. NKX2-1 and p53 in whole lysates of both cells were evaluated by western blotting. β-actin was used as a loading control for whole cell lysates. The cells were trypsinised and collected from the dishes. Samples consisting of 1×10^4^ cells were seeded into Boyden chambers to evaluate the invasion capability, and 1000 cells were seeded in 6-well plates with low-melting agarose gel (0.4% upper agarose gel and 1% bottom agarose gel) for 3 weeks to evaluate soft agar growth efficacy (colonies larger than 100 μm diameters were quantified, as shown black bar=100 μm).(**B**) NKX2-1 suppresses lung nodule formation in wild-type p53 cells, but promotes it in mutated p53 cells. The lung tumour nodules of each group of nude mice which were respectively injected with A549/VC, A549/NKX2-1-OV, TL-13/NC, and TL-13/shNKX2-1 stable clones were counted. Data are presented as means ± SEMs; data were compared between groups using the *t*-test, and *P < 0.05 was considered to be statistically significant.

We examined the possibility that NKX2-1-mediated p53 could modulate soft-agar growth, invasion, and xenograft tumour formation in lung adenocarcinoma cells. NKX2-1 expression vector was transfected into p53-WT A549 and a small hairpin (sh) RNA was used to silence NKX2-1 expression in TL-13 cells. Interestingly, the capabilities of soft-agar growth and invasion were dose-dependently decreased in NKX2-1-overexpressing A549 and H23 cells, and increased in NKX2-1-knockdown TL-4 and TL-13 cells (Figure 3A). The representative soft-agar colonies on soft-agar plates and invasive cells on Matrigel membrane are shown in Figure 3A. The number of lung tumour nodules in nude mice injected with A549 cells with empty vector transfection (VC) was markedly higher than in nude mice injected with NKX2-1-overexpressing A549 stable clone (NKX2-1-OV) (Figure 3B). Conversely, the efficacy of lung tumour nodule formation in nude mice injected with NKX2-1-knockdown TL-13 stable clone was significantly reduced than nude mice injected with TL-13 cells with non-specific shRNA transfection (NC) (Figure 3B). These results clearly indicate that NKX2-1-mediated p53 plays a tumour suppressor property in p53-WT cells, but exhibits an oncogenic role in p53-mutant cells.

### IKKβ/NF-κB activation and EMT formation is responsible for NKX2-1/p53 axis-mediated soft-agar growth and invasiveness

**Figure 4:**
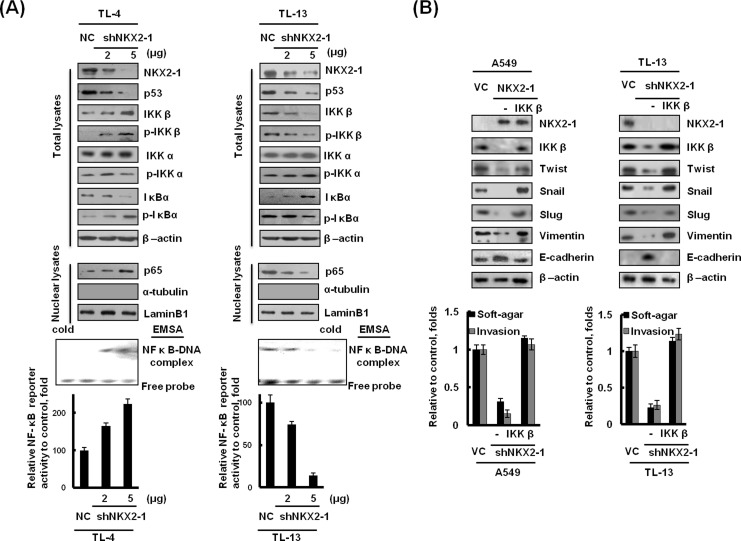
NKX2-1-mediated IKKβ/NF-κB activation promotes soft-agar growth and invasiveness in lung adenocarcinoma cells via induction of EMT (**A**) TL-4 and TL-13 cells were transfected with shNKX2-1 as indicated. The total lysates of NKX2-1, p53, IKKβ, IKKα, p-IKKβ (phosphoY188) and p-IKKα (phosphor T23), p-IκBα (phospho S32), IκBα, and the nuclear fraction of p65 proteins in both cells were evaluated by western blotting. β-actin and Lamin B1 were used as loading controls for total cell lysates and nuclear fractions, respectively (upper panel). Whole cell lysates were evaluated for NF-κB reporter activity by luciferase reporter analysis (lower panel). The DNA-binding capacity of NF-κB in nuclear lysates from TL-4 and TL-13 cells was evaluated by EMSA. (**B**) After a 48-hr transfection of wild-type p53 A549 cells and mutated p53 TL-13 cells with NKX2-1, shNKX2-1, and IKKβ indication. The cells were trypsinised and collected from the dishes. Samples consisting of 1×10^4^ cells were seeded into Boyden chambers to evaluate the invasion capability, and 1000 cells were seeded in 6-well plates with low-melting agarose gel (0.4% upper agarose gel and 1% bottom agarose gel) for 3 weeks to evaluate soft agar growth efficacy (colonies larger than 100 μm diameters were quantified). NKX2-1, IKKβ, Twist, Snail, Vimentin and E-cadherin in whole lysates of both cells were evaluated by western blotting. β-actin was used as a loading control for whole cell lysates.

As mentioned above, NF-κB activation by p53 alteration plays a crucial role in lung adenocarcinoma progression. We therefore explored the possibility that soft-agar growth and invasiveness changed by the NKX2-1/p53 axis could be predominately through modulating IKKβ-mediated p65 nuclear localization. Intriguingly, the expression of IKKβ and phosphorylated (p)-IKKβ were dose-dependently increased in NKX2-1-knockdown TL-4 cells, but decreased in NKX2-1-knockdown TL-13 cells. However, no change of IKKα and p-IKKα expression was observed in NKX2-1-knockdown both cells. IκBα and nuclear p65 expression levels were concomitantly increased NKX2-1-knockdown TL-4 cells, but decreased in NKX2-1-knockdown TL-13 cells (Figure 4A upper panel). The change of NF-κB DNA binding activity and its reporter activity were further confirmed by EMSA and luciferase reporter assay (Figure 4A lower panel). Interestingly, EMT-related genes - Twist, Snail, Vimentin, and Slug were decreased in NKX2-1-overexpressing A549 and NKX2-1-knockdown TL-13 cells; however, the four gene expressions were restored by ectopic IKKβ expression in NKX2-1-overexpressing A549 cells and NKX2-1-knockdown TL-13 cells (Figure 4B, upper panel). Conversely, E-cadherin expression elevated markedly in NKX2-1-overexpressing A549 and NKX2-1–knockdown TL-13 cells compared with their VC and NC cells; however, E-cadherin expression was reduced by ectopic IKKβ expression in NKX2-1-overexpressing A549 and NKX2-1-knockdown TL-13 cells (Figure 4B, upper panel). The changes of soft-agar growth and invasiveness by the NKX2-1/p53 axis are dependent on E-cadherin expression via modulating IKKβ and nuclear p65 expression in both cell types (Figure 4B lower panel). These results clearly indicate that EMT formation due to modulating IKKβ and nuclear p65 expression is responsible for the NKX2-1/p53 axis-mediated soft-agar growth and invasiveness in lung adenocarcinoma cells.

### IKKβ expression modulating by the NKX2-1/p53 axis is at transcriptional level

**Figure 5:**
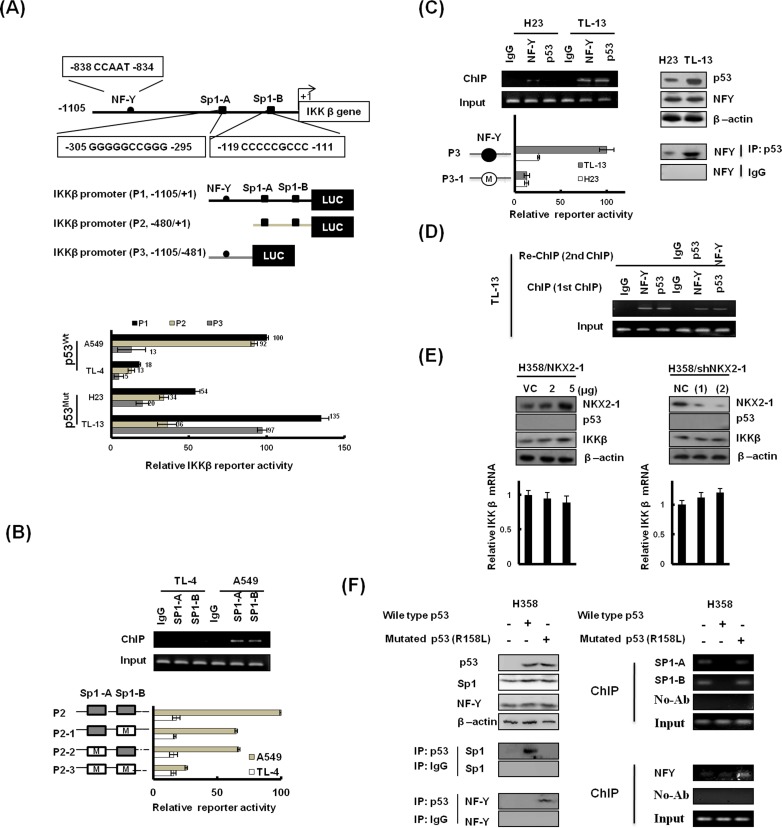
IKKβ expression modulated by NKX2-1-mediated p53 at transcriptional level is dependent on p53 status (**A**) Schematic diagram of IKKβ promoter-driven luciferase reporters: IKKβ (P1, −1105/+1)-Luc, IKKβ (P2, −480/+1)-Luc and IKKβ (P3, −1105/−480)-Luc. Sp1-A and Sp1-B possess putative Sp1 binding sites. (−838 CCAAT −834) possesses NF-Y binding site. The three promoter constructs were co-transfected into A549, TL-4, H23, and TL-13 cells for luciferase reporter assay after 48 hr. (**B**) The binding activity of Sp1 to the IKKβ promoter was evaluated by ChIP in A549 and TL-4. Chromatin was isolated and then immunoprecipitated with Sp1-specific antibody. The two Sp1 putative binding sites on the IKKβ (−480/+1)-Luc were mutated by site-directed mutagenesis, and the mutated promoters were transfected into A549 and TL-4 cells to evaluate reporter activity. (**C**) The binding activity of NF-Y to the IKKβ promoter was evaluated by ChIP in H23 and TL-13. Chromatin was isolated and then immunoprecipitated with NF-Y-specific antibody. The NF-Y putative binding site on the IKKβ (P3, −1105/−480)-Luc was mutated by site-directed mutagenesis, and the mutated promoters were transfected into H23 and TL-13 cells to evaluate reporter activity. IP and western blotting showed immunoprecipitation of p53 with NF-Y. Antibodies used for immunoprecipitation (IP) and western blotting (WB) are designated. (**D**) Re-ChIP assays: In the first ChIP, the chromatin was pulled down with IgG, NF-YA and p53 antibodies, as marked horizontally at the top of the gels. In the second ChIP, the pulled-down chromatin was subsequently interacted with IgG, p53, and NF-Y antibodies, as marked horizontally in the right margin. (**E**) Lysates from H358 cells transfected with NKX2-1 overexpression vector and NKX2-1-knockdown vector were prepared 48 hr after transfection. NKX2-1 and IKKβ were detected by immunoblotting with antibodies. Quantitative real-time PCR amplification of IKKβ on total cellular RNA extracted. β-actin was used as a loading control for whole cell lysates. (**F**) Western blotting and ChIP assays were used to evaluate p53 protein levels and the binding activity of Sp1 and NF-Y onto the IKKβ promoter in p53-deficient H358 cells, which were transiently transfected with WT-p53 or R158L-p53. These lysates were immunoprecipitated with anti p53–conjugated beads. The immunoprecipitates were analyzed by SDS-PAGE, followed by immunoblotting with anti-Sp1 and anti-NFY antibodies.

We recently reported that NKX2-1 was interacted with Sp1 to control IKKβ expression [16]. We therefore explored the possibility that IKKβ expression modulated by the NKX2-1/p53 axis could be regulated at transcriptional level. Three promoters of IKKβ (P1: −1105/+1; P2: −480/+1; and P3: −1105/−481) were constructed to test the hypothesis (http://www.cbrc.jp/research/db/TFSEARCH; Figure 4A upper panel). The IKKβ P1, IKKβ P2, and IKKβ P3 promoter activity was lower in high-NKX2-1 expressing TL-4 cells than in low-NKX2-1 expressing A549 cells (Figure 5A lower panel). The binding activity of Sp1 to the IKKβ P2 promoter was significantly higher in low-NKX2-1 expressing A549 cells than in high-NKX2-1 expressing TL-4 cells (Figure 5B upper panel). The IKKβ P2 promoter activity in A549 cells decreased markedly in Sp1-mutated IKKβ P2 promoters (P2-1, P2-2, and P2-3); however, the decrease in IKKβ P2 promoter activity was not seen in TL-4 cells (Figure 5B lower panel). These results clearly indicate that NKX2-1-mediated p53 down-regulates IKKβ transcription in p53 wild-type cells via decreased Sp1 binding to the IKKβ promoter.

In p53-mutant cells, the IKKβ P3 promoter activity was 0.72 folds of IKKβ P1 promoter activity, and 2.69 folds of IKKβ P2 promoter activity in high-NKX2-1 expressing TL-13 cells; however, the IKKβ P3 promoter activity was 0.37 folds of the IKKβ P1 promoter activity and 0.58 folds of the IKKβ P2 promoter activity in low-NKX2-1 expressing H23 cells (Figure 4A lower panel). The binding activity of NF-Y and p53 to IKKβ P3 promoter was significantly higher in TL-13 cells than in H23 cells (Figure 4C left panel). IKKβ P3 promoter activity decreased markedly in TL-13 cells, but the decrease of IKKβ P3 promoter activity was not observed in H23 cells when the putative binding site of NF-Y to the P3 promoter was mutated by site-directed mutagenesis (Figure 5C lower panel). In addition, p53 expression was higher in TL-13 cells than in H23 cells, but similar expression levels of NF-Y was observed in both cells (Figure 4C right upper panel). The interaction of NF-Y with mutant p53, evaluated by immunoprecipitation (IP), was more pronounced in TL-13 cells than in H23 cells (Figure 5C right lower panel). ChIP and re-ChIP analysis confirmed that NF-Y, mutant p53, and mutant p53/NF-Y complex were indeed bound to the IKKβ promoter in high-NKX2-1 expressing TL-13 cells (Figure 5D). As shown in Figure 5E, IKKβ expression level was not changed by NKX2-1-overexpression and -knockdown in p53-null H358 cells. These results clearly indicate that increased mutant p53/NF-Y complex by NKX2-1-mediated p53 is responsible for up-regulation of IKKβ transcription in p53-mutant cells. Collectively, IKKβ expression modulated by NKX2-1-mediated p53 at transcriptional level is dependent on p53 status.

To explore how wild-type or mutant p53 regulates IKKβ expression, immunoprecipitation (IP) analysis was performed to examine the possibility that wild-type or mutant p53 could differently interact with Sp1 and NF-Y to regulate IKKβ transcription. As shown in Figure 5F, Sp1 was indeed interacted with wild-type p53, not with mutant p53 in H358 cells transfected with wild-type or mutant p53 (R158I). However, NF-Y was only bound with mutant p53, not with wild-type p53 in H358 cells (left panel). ChIP analysis further confirmed that the binding of Sp1 to the IKKβ promoter was diminished by wild-type p53, not by mutant p53 transfection; however, the binding of NF-Y to the IKKβ promoter was only revealed in mutant p53-transfected H358 cells (right panel). These results clearly indicate that Sp1 and NF-Y is differentially bound to the IKKβ promoter via modulated by wild-type p53 and mutant p53.

**Figure 6:**
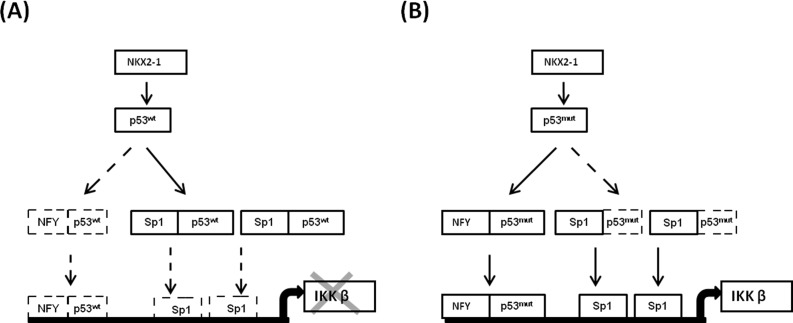
The possible mechanism of the NKX2-1/p53 axis on regulation of IKKβ transcription via modulating Sp1 and NF-Y binding to IKKβ promoter

We further examined the role of the NKX2-1/p53/Sp1 cascade in the regulation of IKKβ transcription. As shown in Supplementary Figure 1, IKKβ mRNA expression was most increased by transfected with R158L p53 mutant plasmid, followed by NKX2-1-knockdown or p53-knockdown; however, the increase of IKKβ mRNA expression was rescued by p53-WT transfection or Sp1 silencing in TL-4 cells. The most increase in IKKβ expression was observed in A549 cells transfected with R158L p53 mutant plasmid; however the IKKβ expression in A549 cells was markedly decreased by NKX2-1 or p53-WT overexpression or Sp1 silencing. Conversely, the IKKβ expression was greatest increased by NKX2-1 overexpression or M246I p53 mutant transfection, but the increase of IKKβ expression was restored by NF-Y silencing or p53-mutant knockdown in H23 cells. IKKβ expression was markedly decreased by NKX2-1 knockdown, NF-Y silencing, p53-mutant silencing, and NKX-2-1 knockdown plus p53-WT overexpression when compared with TL-13 VC cells. Interestingly, the decrease of IKKβ expression in NKX2-1-knockdown TL-13 cells was reversed by p53-mutant R158L transfection. To test whether different mutant p53 could have different effects on IKKβ transcription, four different p53 mutant expression vectors (E286Q, S240R, M246I, and R158L) were transfected in p53 null H358 cells. ChIP analysis indicated that the binding activity of the mutant p53 M246I and R158L was greater than that of p53 mutant E286Q and S240R. The binding activity of NF-Y on IKKβ promoter was only observed in H358 cells with p53 mutant M246I and R158L transfection. The reporter activity of IKKβ evaluated by luciferase reporter assay was greater increased by p53 mutant M246I and R158L transfection in H358 cells when compared with p53 mutant E286Q and S240R transfection. These results clearly indicate that the NKX2-1/p53/Sp1 cascade plays a critical role in regulation of IKKβ transcription. The possible route of the NKX2-1/p53 axis on regulation of IKKβ transcription via modulating Sp1 and NF-Y binding to IKKβ promoter is proposed in Figure 6.

### IKKβ mRNA was negatively correlated with NKX2-1 mRNA expression in p53-WT patients, but positively associated with NKX2-1 mRNA expression in p53-mutant patients

We examined the possibility that NKX2-1 expression could be associated with IKKβ expression in 157 tumours from lung adenocarcinoma patients. NKX2-1 and IKKβ mRNA expression levels were evaluated by real-time RT-PCR. As shown in Table 1, NKX2-1 mRNA expression was not associated with IKKβ mRNA; intriguingly, NKX2-1 mRNA was positively correlated with p53 mRNA expression in all studied population, but the prevalence of high-IKKβ mRNA expression was more common in p53-mutant patients than in p53-WT patients (P = 0.035). However, p53 mRNA expression was not associated with the p53 status, IKKβ mRNA, IKKβ protein, and p65 nuclear translocation. Being stratified the p53 status, NKX2-1 mRNA expression levels were negatively correlated with p53 mRNA expression in p53-WT patients (P < 0.001), but the positive correlation between IKKβ and p53 mRNA expression was observed in p53-mutant patients (P = 0.022, Table 1). Interestingly, low-IKKβ and low-p53 mRNA tumours was more frequently observed in p53-WT patients (P = 0.016 for IKKβ; P = 0.017 for p53), but the opposite was seen in p53-mutant patients (P = 0.002 for IKKβ; P = 0.003 for p53). Immunohistochemistry analysis showed that IKKβ mRNA expression was positively correlated with its protein expression and nuclear p65 expression in a subset of the studied population (87 of 157), (P < 0.001 for IKKβ immunostaining and nuclear p65 expression; Table 1). These results obtained from lung adenocarcinoma patients appear to support the mechanistic findings of the cell models.

**Table 1:** The association of IKKβ and p53 mRNA levels with p53 mutational status, IKKβ protein, p65 nuclear translocation, and NKX2-1 mRNA in tumors from lung adenocarcinoma patients

Parameters		IKKβ mRNA^b^		*P* value	p53 mRNA^b^		*P* value
	No.	Low (%)	High (%)		Low (%)	High (%)	
**p53 mutational status**							
No	82	47 (60)	35 (44)	0.045	39 (50)	43 (54)	0.578
Yes	75	31 (40)	44 (56)		39 (50)	36 (46)	
**IKKβ^c^**							
Low	45	31 (72)	14 (31)	<0.001	19 (45)	26 (57)	0.242
High	42	12 (28)	30 (69)		23 (55)	19 (43)	
**P65 translocation^c^**							
Cytoplasm	40	31 (72)	9 (20)	<0.001	19 (45)	21 (46)	0.894
Nucleus	47	12 (28)	35 (80)		23 (55)	24 (54)	
**NKX2-1 mRNA^b^**							
Low	78	41 (52)	37 (46)	0.473	54 (69)	24 (30)	<0.001
High	79	37 (48)	42 (54)		24 (31)	55 (70)	
**IKKβ mRNA^b^**							
Low	78				38 (48)	40 (51)	0.810
High	79				40 (52)	39 (49)	
**P53-WT**							
**NKX2-1 mRNA**							
Low	46	21 (45)	25 (71)	0.016	31 (79)	15 (34)	<0.001
High	36	26 (55)	10 (29)		8 (21)	28 (66)	
**p53 mRNA^b^**							
Low	39	17 (36)	22 (62)	0.017			
High	43	30 (64)	13 (38)				
**P53-mutant**							
**NKX2-1 mRNA**							
Low	32	20 (64)	12 (27)	0.002	23 (58)	9 (25)	0.003
High	43	11 (36)	32 (73)		16 (42)	27 (75)	
**p53 mRNA^b^**							
Low	39	21 (67)	18 (40)	0.022			
High	36	10 (33)	26 (60)				

P values are calculated by Chi-square test.

b Median value of NKX2-1, p53 and IKKβ mRNA expression levels in lung tumors was used as a cutoff point to divide tumors into “low” and “high” category.

c Immunohistochemical assay

Immunostaining score: [proportion score (0 = 0/100; 1 = 1/100~1/10; 2 = 1/10~1/3; 3 = 1/3~2/3; 4 = 2/3~1; and 5 = 100/100) + intensity score (0 = negative; 1 = weak; 2 = intermediate; and 3 = strong)]. The score are ranged from 0 to 8 and the score of 4 was used as the cutoff point to divide tumors into as “low” and “high” immunostaining.

### High NKX2-1 mRNA level was associated with favourable OS and RFS in p53-WT patients, but unfavourable OS and RFS was observed in p53-mutant patients with high NKX2-1 mRNA levels

We next examined whether IKKβ expression modulated by the NKX2-1/p53 axis could be associated with overall survival (OS) and relapse free survival (RFS) in lung adenocarcinoma. The prognostic value of p53 status evaluated by Cox regression analysis was not observed in this study population (Table 2). However, patients with high-IKKβ mRNA, high-IKKβ protein, and nuclear p65 tumours exhibited poorer OS and RFS than those with low-IKKβ mRNA, low-IKKβ protein, and cytoplasmic p65 tumours (OS: HR, 1.93, P = 0.001 for IKKβ mRNA; HR, 2.68, P = 0.008 for IKKβ protein; HR, 3.24, P = 0.003 for nuclear p65; RFS: 1.91, P = 0.004 for IKKβ mRNA; HR, 2.01, P = 0.017 for IKKβ protein; HR, 2.14, P = 0.010 for nuclear p65; Table 2). However, the prognostic value of p53 mRNA was not observed in this all study population. The median survival months for OS and RFS in patients with high-IKKβ mRNA, high-IKKβ protein, and nuclear p65 tumours were shorter than their counterparts (Table 2). Interestingly, p53-WT patients with high-NKX2-1 mRNA and high-p53 mRNA tumours exhibited more favourable OS and RFS compared with those with low-NKX2-1 mRNA tumours (NKX2-1: HR, 0.48, P = 0.022 for OS; HR, 0.40, P = 0.003 for RFS; p53: HR, 0.43, P = 0.009 for OS; HR, 0.42, P = 0.003 for RFS); however, p53-mutant patients with high-NKX2-1 mRNA and high-p53 mRNA tumours had more unfavourable OS and RFS than those with low-NKX2-1 mRNA tumours (NKX2-1: HR, 7.41, P = 0.001 for OS, HR, 2.71, P = 0.010 for RFS; p53: HR, 3.74, P = 0.004 for OS; HR, 2.13, P = 0.032 for RFS; Table 2). The reverse prognostic value of NKX2-1 mRNA expression on OS and RFS between p53-WT and p53-mutant patients may support the findings of cells and animal models.

**Table 2:** Cox regression analysis of various potential factors on OS and RFS in lung adenocarcinoma patients

	No.	OS			RFS		
		Median survival, (months)	HR^a^ 95% CI	*P* value	Median survival, (months)	HR^a^ 95% CI	*P* value
** p53 status**							
P53-WT	82	18.0	1	0.138	17.2	1	0.423
P53-mutant	75	33.1	0.69 (0.42-1.13)		29.7	0.84 (0.55-1.29)	
**IKKβ mRNA^b^**							
Low	78	30.3	1	0.011	26.4	1	0.004
High	79	16.9	1.93 (1.16-3.19)		14.0	1.91 (1.23-2.96)	
IKKβ^c^							
Low	45	28.9	1	0.008	25.4	1	0.017
High	42	18.1	2.68 (1.19-5.29)		14.5	2.01 (1.13-3.55)	
**P65** **translocation^c^**							
Cytoplasm	40	29.6	1	0.003	26.6	1	0.010
Nucleus	47	15.9	3.24 (1.48-7.06)		14.1	2.14 (1.20-3.85)	
**NKX2-1 mRNA^b^**							
Low	78	17.5	1	0.767	14.1	1	0.670
High	79	24.0	1.07 (0.67-1.71)		24.0	0.91 (0.60-1.38)	
**p53 mRNA^b^**							
Low	78	21.6	1	0.729	18.3	1	0.393
High	79	22.9	0.91 (0.57-1.48)		22.2	0.83 (0.54-1.27)	
**P53-WT** **NKX2-1 mRNA**							
Low	46	22.2	1	0.022	22.2	1	0.003
High	36	14.0	0.48 (0.25-0.90)		14.1	0.40 (0.22-0.73)	
**p53 mRNA^b^**							
Low	39	17.5	1	0.009		1	0.003
High	43	22.2	0.43 (0.22-0.80)			0.42 (0.23-0.75)	
**P53-mutant** **NKX2-1 mRNA**							
Low	32	43.2	1	0.001	30.6	1	0.010
High	43	30.6	7.41 (2.28-24.08)		24.1	2.71 (1.26-5.81)	
**p53 mRNA^b^**							
Low	39	43.8	1	0.004		1	0.032
High	36	23.4	3.74 (1.52-9.20)			2.13 (1.06-4.26)	

a HR was adjusted by the parameters including age, gender, smoke and stage. OS: overall survival. RFS: relapse free survival.

b Median value of NKX2-1, p53 and IKKβ mRNA expression in lung tumors was used as a cutoff point to divide tumors into “low” and “high” category.

c Immunohistochemistry analysis.

## DISCUSSION

In the present study, we provide evidence that IKKβ expression modulated by the NKX2-1/p53 axis at transcription level is responsible tumour progression in lung adenocarcinoma. The IKKβ-mediated p65 nuclear localization is essential for promoting inflammatory-associated cancer, such as lung adenocarcinoma, colon cancer, pancreatic adenocarcinoma, and ovarian cancer [10, 17, 18]. IKKβ-mediated p65 nuclear translocation is little reported via regulation of IKKβ at transcriptional level. In the present study, we demonstrated that upregulation of IKKβ transcription by the NKX2-1/p53 axis may be responsible for p65 nuclear localization and NF-κB activation in lung adenocarcinoma cells (Figure 4). To the best our knowledge, there is for the first time to indicate that upregulation of IKKβ expression at transcription level may activate the NF-κB signalling pathway and consequently to promote tumor progression and metastasis. A recent report using a mouse lung cancer model revealed that IKKβ depletion in tumour cells significantly attenuated tumour proliferation and significantly prolonged mouse survival [12]. An NF-κB pathway-mediated positive feedback loop amplified Ras activity and led to chronic inflammation and precancerous lesions in mice that expressed physiological levels of oncogenic Kras [19]. However, the positive feedback loop of the NF-κB/Ras axis to initiate inflammatory-induced tumour development was able to be blocked by the deletion of IKKβ. Therefore, IKKβ plays a crucial role in Kras-mutated tumour development, such as a large proportion of Kras mutation in colon, pancreas, and lung cancers [19]. In this study population, 4 out of 157 patients were detected to have Kras mutation (2.5%) and this was consistent with a Taiwanese study [20]. Among the four lung adenocarcinoma cells used in this study, TL-13 cells possess wild-type Kras gene, the other three cells harbour Kras mutations. However, the efficacy of soft-agar growth, invasion, and xenograft tumour formation was dependent on the NKX2-1/p53 axis-modulated IKKβ expression, not dependent on Kras mutation. In addition, the NKX2-1/p53 axis-modulated IKKβ expression was associated with poorer outcome in lung adenocarcinoma patients. Takahashi's group pointed out NKX2-1 as a double-edged sword for cancer cell survival and progression. For example, NKX2-1 plays lineage-survival oncogene in lung adenocarcinoma and enhanced EFGR-driven lung tumorigenesis. Conversely, NKX2-1 acts suppressor role to reduce invasion and metastasis and Kras-driven lung tumorigenesis [21]. Here, we present a novel mechanism to indicate that lung adenocarcinoma progression mediated by the NKX2-1/p53 axis-modulated IKKβ expression is dependent on p53 status, particularly in wild-type Kras lung adenocarcinoma.

Cross-talk between p53 and NF-κB pathway has been reported [22, 23]. Functional inactivation of p53 and constitutive activation of the NF-κB signalling pathway have been associated with several human cancers [24]. Most studies regarding how the cross-talk between p53 and NF-κB pathway promotes tumour growth investigated at the protein level, not at the transcriptional level [25-27]. The IKKβ/NF-κB pathway is required for the development of lung adenocarcinoma in a mouse model, in which a concomitant loss of p53 and expression of oncogenic Kras (G12D) resulted in NF-κB activation [28]. However, p53 restoration led to the inhibition of the ability of NF-κB to induce apoptosis in p53-null lung cancer cell lines [29, 30]. Therefore, NF-κB performs a critical signalling function in lung tumour development, and this requirement depends on p53 status.

In summary, we provided evidence to demonstrate that NKX2-1/p53 axis-modulated IKKβ-mediated p65 nuclear localization plays a critical role in tumour progression of lung adenocarcinoma. We therefore suggest that IKKβ inhibitor might have the potential to improve tumour regression and outcomes in p53-mutant lung adenocarcinoma patients with high-NKX2-1 tumours or p53-WT lung adenocarcinoma patients with low-NKX2-1 tumours.

## MATERIALS AND METHODS

### Study subjects

Lung tumors were enrolled from 157 lung adenocarcinoma patients who were received the surgical therapy at the Division of Thoracic Surgery, Taichung Veterans General Hospital, Taiwan, between 1993 and 2004. This study is approved by the Institutional Review Board, Taipei Medical University Hospital (TMUH No: 201301051). The tumor type and stage of each collected specimen were histologically determined according to the WHO classification system. Cancer relapse data were obtained by chart review and further confirmed by two clinical physicians.

### Cell lines

A549, H23, H358, H441, H1355 and H1975 lung adenocarcinoma cells were obtained from the American Type Culture Collection (ATCC) and cultured as described previously [31]. CL-3, CL1-0 and CL1-5 cells were kindly provided by Dr. P. C. Yang (Department of Internal Medicine, National Taiwan University Hospital, Taipei, Taiwan). TL-4, TL-5, TL-6 TL-10 and TL-13 lung adenocarcinoma cells were established from pleural effusions from Taiwanese lung cancer patients and cultured as described previously [32, 33].

### Quantitative real-time reverse transcriptase (RT)-polymerase chain reaction (PCR)

Target gene mRNA expressions were performed by real-time RT-PCR assay. 18S rRNA was used as internal control. The relative amounts of the target gene, standardized against the amount of 18S rRNA, were expressed as ΔC_t_ =C_t_ (target) - C_t_ (18S rRNA). The ratio of gene mRNA copies to 18S rRNA copies was then calculated as 2 ^−ΔCt^ × K (K =10^4^, a constant). Primers were listed in Supplementary Table 1.

### Western blotting

Cells were washed twice on ice with PBS before adding protein lysis buffer (1× protease inhibitor cocktail, Roche, Basel, Switzerland, 1.5mM EDTA, 1mM DTT, 10% glycerol, 25mM HEPES, pH 7.6). The protein concentration was determined by the Bradford assay (BioRad, Hercules, CA) using BSA as a standard. Total protein (20μg) was resolved by 10% SDS-PAGE for subsequent western blot analysis using antibodies against the following proteins (diluted in TTBS (Tween–Tris Buffered Saline: 0.02% Tween-20 in 100mM Tris-CL [pH 7.5], 1:1000) as indicated): anti-p53 (DO7, DAKO, Glostrup, Denmark), anti-NKX2-1 (sc-53136, Santa Cruz, Santa Cruz, CA), anti-Sp1 (sc-14027, Santa Cruz, CA), anti-p65 (sc-8008, Santa Cruz, CA), anti-Snail (sc-28199, Santa Cruz, CA), anti –NF-Y (sc-17753, Santa Cruz, CA), anti-vimentin (sc-6260, Santa Cruz, CA), anti-IKKβ (sc-271782, Santa Cruz, CA), ant-α-tubulin (sc-5266, Santa Cruz, CA), anti-p21 (sc- 6246, Santa Cruz, CA) and anti-E-cadherin (cat. 610182, BD Biosciences, Franklin Lakes, NJ). The gel was transferred to a Hybond-C Extra membrane (GE Healthcare, Little Chalfont, UK) and immunoblotted with primary antibody, as indicated in the figure legends. Anti-mouse or rabbit IgG conjugated to horseradish peroxidase was used as the secondary antibody for detection using an ECL western blot detection system.

### Plasmid construction

Full-length human NKX2-1 cDNA was amplified from TL-4 mRNA by the RT-PCR using primers based on published mRNA sequences. The NKX2-1 cDNA was cloned into pcDNA3.1 Zeo(+) (Invitrogen, Carlsbad, CA). The wild-type p53 plasmids were kindly provided by Dr. JL Ko from the Institute of Medicine at Chung Shan Medical University. The shNKX2-1 (GCCAAGGTTAGAACCTGCAAA; AGTCCAGTAAC CGGGAATATG), plasmids were obtained from the National RNAi Core Facility located at the Institute of Molecular Biology/Genomics Research Center, Academia Sinica, Taiwan. The IKKβ-Luc plasmids were constructed by inserting KpnI/XhoI fragments into a KpnI/XhoI-treated pGL3 vector (Promega, Madison, WI).

### Flow cytometry analysis

For flow cytometry analysis, cells (5 ×10^5^) were seeded onto 6 cm dishes for 24 hours. The cultured cells were then harvested and centrifuged at 200 g for 5 minutes. The cell pellets were washed twice with 1 × PBS and fixed with 70% ethanol at 4 °C for 30 minutes. After centrifugation at 200 g for 5 minutes, the cell pellets were washed with 1 × PBS to remove any residual ethanol. Finally, the cells were resuspended in 1 mL of solution containing 0.5 mg/mL RNase A, 1% Triton X-100, and 40 μg/mL propidium iodide, and incubated at 37 °C for 30 minutes. The cells were filtered through a 40 μm nylon mesh before flow cytometry analysis of cell cycle distribution using a FACS-Calibur flow cytometer (Becton Dickinson, Franklin Lakes, NJ USA).

### Site-directed mutagenesis

Site-directed mutagenesis was performed to generate the mutant p53, Sp1 binding sites of IKKβ and NKX2-1 binding sites of p53 promoter constructs using the complementary oligos (Supplementary Table 1). Plasmids contain multiple point mutations of the sites were generated using the Quick Change site-directed mutagenesis system (Stratagene, Santa Clara, CA). Different concentrations of the expression plasmids were transiently transfected into lung cancer cells (1 × 10^6^) using the Transfast reagent (Thermo, Waltham, MA). After 48 hours, the cells were harvested, and whole-cell extracts were assayed in subsequent experiments.

### Luciferase reporter assay

Luciferase assays were conducted using the luciferase reporter assay system (Promega, Madison, WI) 48 hours after transfection. Normalized luciferase activity was reported as luciferase activity/β-galactosidase activity.

### Nuclear/cytoplasmic fraction of lung cancer cells

Nuclear/cytoplasmic lysates were prepared using a nonionic detergent method. Briefly, nuclear extracts were prepared in extraction buffer (10 mM KCl, 10 mM HEPES, pH 7.9, 1.5 mM MgCl_2_, and 0.5 mM dithiothreitol) and protease inhibitors. The extracts were centrifuged at 14,000 g for 1 min and the supernatant was used as the cytoplasmic extract and placed into new tubes. The pellet was suspended with extraction buffer (420 mM NaCl, 20 mM HEPES, pH 7.9, 1.5 mM MgCl2, 0.2 mM EDTA, 25% glycerol, 0.5 mM dithiothreitol, and 0.5 mM PMSF) and centrifuged again at 14,000 g for 5 min. The supernatant fraction was used as the nuclear extract. Both extracts were stored at −70°C for further experiments.

### Chromatin immunoprecipitation (ChIP) assays

ChIP analysis was performed according to a published procedure, with the following modifications. Immunoprecipitated DNA was ethanol precipitated and resuspended in 20 μl ddH_2_O. Total input samples were resuspended in 100 μl ddH_2_O and diluted 1:100 before PCR analysis. PCR amplification of immunoprecipitated DNA was carried out with diluted aliquots using the oligonucleotides as primers. The PCR products were separated on 2% agarose gels and analyzed by ethidium bromide staining. All ChIP assays were performed at least twice and produced similar results.

### Electrophoretic mobility shift assay (EMSA)

Preparation 10 pmol biotin-labeled oligonucleotide of NF-κB binding sites which added to the nuclear extracts (10 μg). The reaction was incubated at 25°C for 20 min with The sequence was as follows: 5′CAGTGGAATTCCCCAGCC3′. The reaction mixture was electrophoresed at 4°C in 6% polyacrylamide gel using TBE running buffer. The detecting solution was purchased from Thermo (Waltham, MA), and the protocols were performed according to the instructions of the manufacturer.

### Xenograft tumor formation

The NC (shGFP), shNKX2-1, VC (pcDNA3.1 Zeo(+)), NKX2-1 and IKKβ plasmids (3 μg) were mixed with TransFast transfection reagent (Promega, Madison, WI) and added to 1 × 10^5^ A549 and TL-13 cells, respectively. After 24 h, stable transfectants were selected using 2 μg/ml puromycin. The selection medium was replaced every 3 days for 3 weeks. Gene expression was confirmed by western blot and RT-PCR analyses. Female immunodeficient nude mice (BALB/c nu/nu mice) 5 weeks old and weighing 18–22g, were injected with phosphate-buffered saline (PBS), A540/VC, A549/NKX2-1, TL-13/NC, and TL-13/shNKX2-1 cells via the tail vein (10^6^ cells in 0.1 mL of PBS). After 2 months, mice were sacrificed by an overdose with anesthetic carbon dioxide. The number of metastatic lung tumor nodules was counted under a dissecting microscope.

### Statistical analysis

The Student's test and Chi-square test were applied for data analysis performed using SPSS software (Version 13.0 SPSS Inc., Chicago, IL). For survival data, statistical differences were calculated by the log-rank test. Survival curves were plotted using the Kaplan-Meier method and variables related to survival were analyzed using Cox's proportional hazards regression model with SPSS software. P < 0.05 was considered to be statistically significant.

## SUPPLEMENTARY MATERIAL, FIGURES AND TABLE


