# Supplementary material for: NKX2-1-mediated p53 expression modulates lung adenocarcinoma progression via modulating IKKβ/NF-κB activation
Source: Oncotarget. 2015 Mar 30;6(16):14274–89. doi: 10.18632/oncotarget.3695 (PMC4546466; doi:10.18632/oncotarget.3695)
Supplement: Supplementary file 1 [file oncotarget-06-14274-s001.pdf]

NKX2-1-mediated p53 expression modulates lung adenocarcinoma progression via modulating IKKβ/NF-κB activation

Supplementary Material

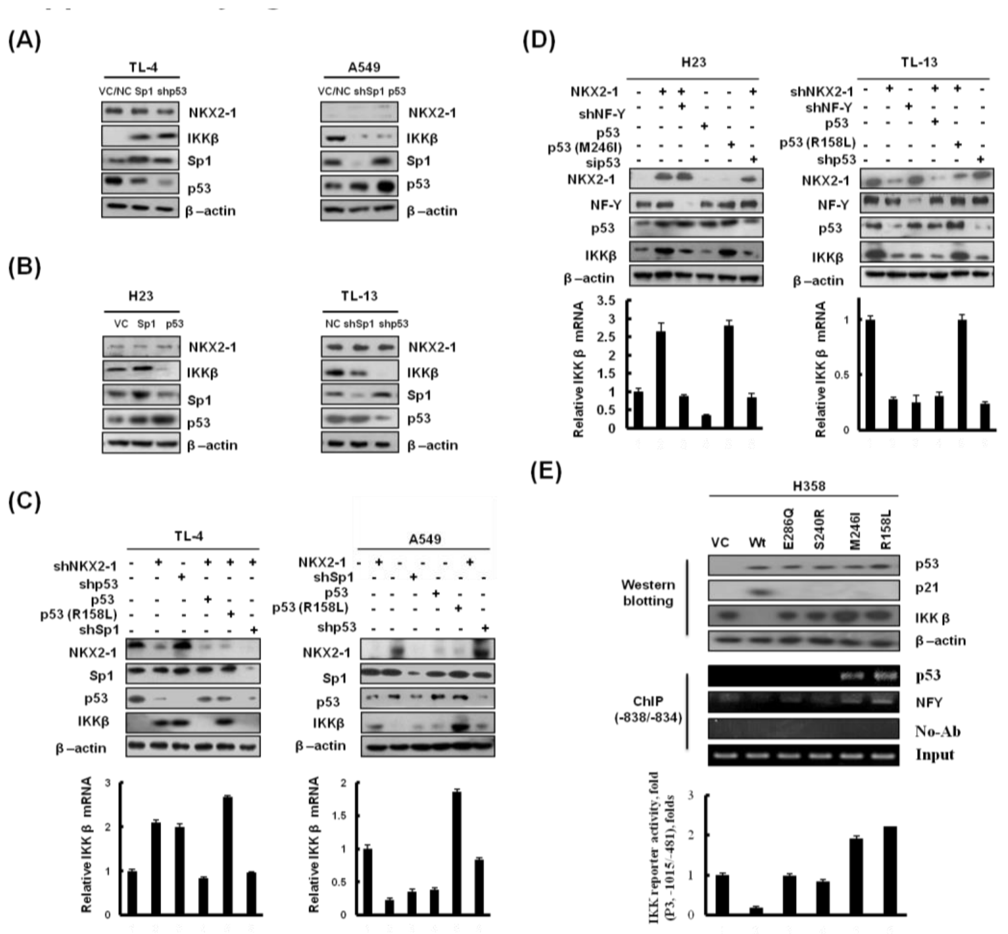

Supplementary Figure 1: IKKβ transcription is regulated by the NKX2-1/p53/Sp1 cascade in lung adenocarcinoma cells. (A, B) TL-4 and H23 cells were overexpressed with Sp1 expression vector or knocked down by shp53 and A549 and TL-13 cells were transfected with shSp1 or overexpressed with p53 for 48 hr and then the change of IKKβ, NKX2-1, Sp1, and p53 expression was evaluated by western blotting. (C) TL-4 cells were transfected with shNKX2-1 or shp53 and/or combined with WT-p53, mutant p53 R158L or shSp1 transfection, and A549 cells were transfected with NKX2-1 or shSp1, WT-p53, mutant p53 R158L or Shp53. After 48 hr, the cell lysates

were prepared for extraction of total proteins to evaluate the expression of NKX2-1, Sp1, p53, and IKKβ by western blotting analysis. β-catenin was used as protein loading controls. IKKβ mRNA expression levels in both cell types with different treatments were determined by real-time RT-PCR. The relative of IKKβ mRNA expression levels of TL-4 and A549 cells with different treatments was referenced by both control cells as “1”. (D) H23 cells were transfected with NKX2-1 and/or combined with shNF-Y, WT-p53, mutant p53 M246I, or shp53 and TL-13 cells were transfected with shNKX2-1 and/or combined with shNF-Y, WT-p53, mutant p53 R158I, and shp53. The total proteins of cell lysates were used to determine the expression of NKX2-1, NF-Y, p53, and IKKβ by western blotting. The total RNAs of cell lysates were used to determine IKKβ mRNA expression levels. (E) p53 null H358 cells were transfected with WT-p53, mutant p53 E286Q, S240R, M246I, or R158L and then evaluate the expression of p53, p21, and IKKβ by western blotting. B-catenin was used as a protein loading control. The binding activity of WT-p53 and different mutant p53 on the IKKβ promoter (-838/-843) was evaluated by ChIP analysis. Luciferase reporter activity assay was performed to detect the IKKβ reporter activity in H358 cells with WT-p53 or different mutant p53 transfections. The VC cells was used as 1 to relative IKKβ reporter activity in H358 cells with different treatments.

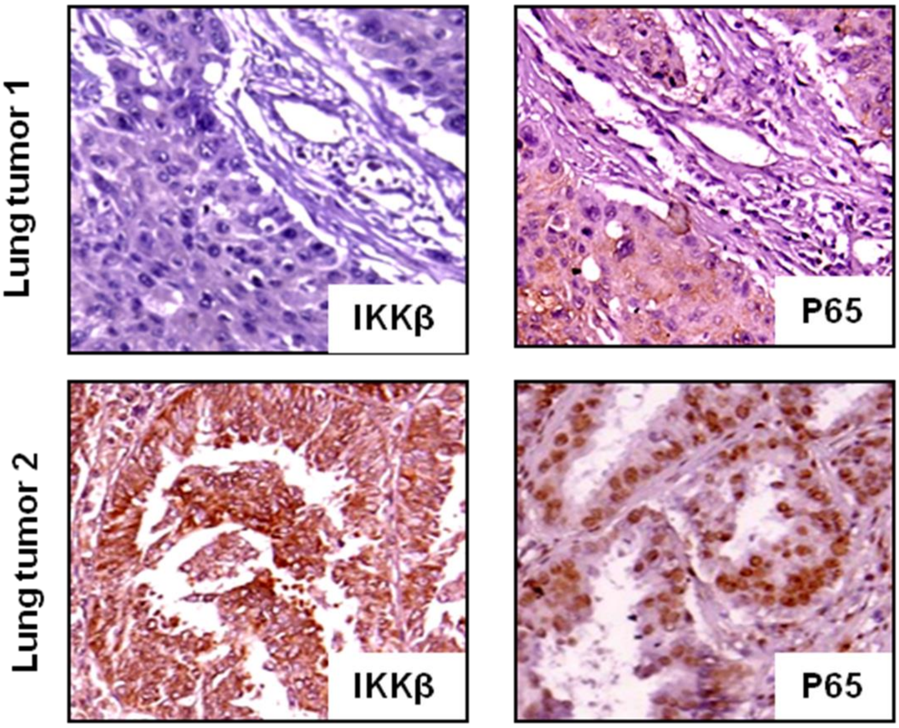

Supplementary Figure 2: Immunohistochemical analysis of paraffin-embedded lung adenocarcinoma, using IKKβ Ab (left). The serial sections were stained for p65 Ab (right). Lung tumor 1: IKKβ, Low; nuclear p65, Low Lung tumor 2: IKKβ, High; nuclear p65, High

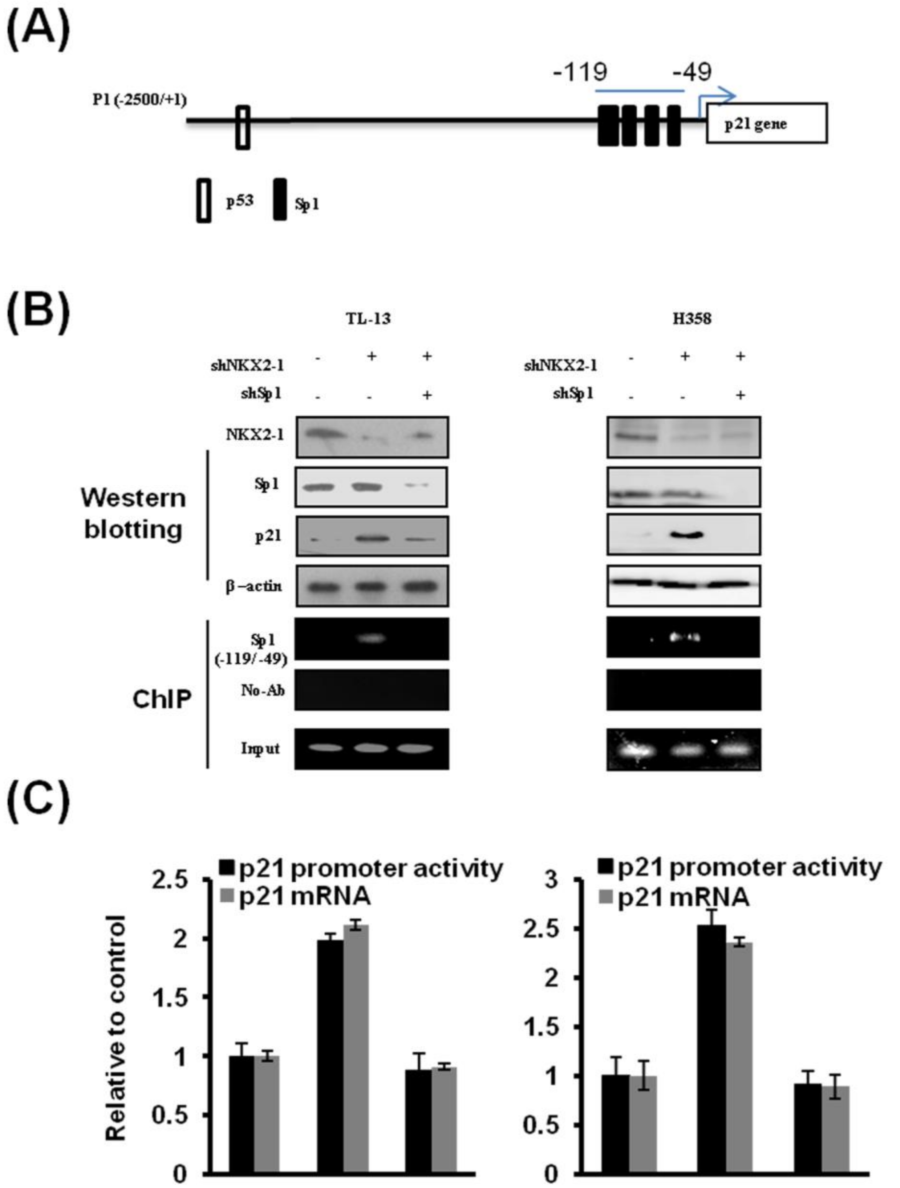

Supplementary Figure 3: NKX2-1 suppresses Sp1 binding to the p21 promoter. (A) The binding sites of p53 and Sp1 on the p21 promoter were discovered by a software analysis (http://www.cbrc.jp/research/db/TFSEARCH). (B) Western blotting and ChIP assays were used to evaluate p21 protein expression and the binding activity of Sp1 onto the p21 promoter (-119/-49) in p53 mutant TL-13 and p53-deficient H358 cells, which were transiently transfected with shNKX2-1 or shSp1 vectors. (C)

Luciferase reporter activity assay (-2500/+1) and the p21 mRNA level was evaluated by real-time RT-PCR in p53 mutant TL-13 and p53-deficient H358 cells, which were transiently transfected with shNKX2-1 or shSp1 vectors for 48 hr.

Supplementary Table 1:
List of primer sequences and their reaction conditions used in the present study.

NKX2-1 real-time		
	Forward	AGCACACGACTCCGTTCTCA
	Reverse	GCCCACTTTCTTGTAGCTTTCC
IKKβ real-time		
	Forward	CCCTGCCGACAGAGTTAGCA
	Reverse	CTGTCCCAAGGCGCTCTTT
p53 real-time		
	Forward	AGAAAACCTACCAGGGCAGCTA
	Reverse	GGGAGTACGTGCAAGTCACAGA
NKX2-1 CHIP (-1155/-1147)		
	Forward	AGATAGTGCT ATGAG AATG
	Reverse	CATTCTCATA GCACT ATCA
NKX2-1 CHIP (-696/-674)		
	Forward	GCACT AAAGG AGGCT GAG
	Reverse	CTCAG CCTCC TTTAG TGC
Sp1 CHIP (-305/-205)		
	Forward	GAAATTCCACCGAGGTG
	Reverse	CAAGTTACTTCCACCTG
Sp1 CHIP (-121/-111)		
	Forward	AAATCGGTGAGCACGGTC
	Reverse	CTGCTCTGACGTCACGGA
Sp1 complementary oligos (-305/-205)		
	Forward	TGAAAGCCTAGGTTGCCGGGTGAAGAAATC
	Reverse	GATTTCTTCACCCGGCAACCTAGGCTTTCA
Sp1 complementary oligos (-121/-111)		
	Forward	TTGTCTAGAGACCACACGCCACCCTCGCTC
	Reverse	GAGCGAGGGTGGCGTGTGGTCTCAGACAA
NKX2-1 complementary oligos (-1155/-1147)		

	Forward	GCCCT TAGGCCGTGTATATGC
	Reverse	GCATA TACAC GGCCT AAGGG C
NKX2-1 complementary oligos (-696/-674)		
	Forward	ATCTTGGCACCCTAGGAGGCTG
	Reverse	CAGCC TCCTA GGGTG CCAAG AT
NF-Y CHIP (-838/-834)		
	Forward	TGTAG TCAGG AGAAT C
	Reverse	GTGCA GGGCT CTGTT
NF-Y complementary oligos (-838/-834)		
	Forward	GATGCAGTATTTTCCTTTTTACAG
	Reverse	CTGTA AAAAG GAAAA TACTG CATC
